# Supplementary material for: Direct observation of 3D nitrogen distribution in silicon-based dielectrics using atom probe tomography
Source: Nat Commun. 2025 Jul 1;16:5612. doi: 10.1038/s41467-025-60732-2 (PMC12214997; doi:10.1038/s41467-025-60732-2)
Supplement: Supplementary file 1 — Supplementary Information [file 41467_2025_60732_MOESM1_ESM.pdf]

# Direct observation of 3D nitrogen distribution in silicon-based dielectrics using atom probe tomography

Byeong-Gyu Chae[1]*, Jeong Yeon Won[1], Young Sik Shin[1], Dong Jin Yun[1], Jae min Ahn[1], Seon Tae Park[1], Ki-bum Lee[2], Hokyun An[2], Mina Seol[2], I-Jun Ro[3], Se-Ho Kim[3], Chunhyung Chung[2] and Eunha Lee[1]*

[1]Analytical Engineering Group, Material Research Center, Samsung Advanced Institute of Technology, Samsung Electronics Co., Ltd., 130 Samsung-ro, Suwon, 16678, Republic of Korea

[2]Process Development Team, Semiconductor R&D Center, Samsung Electronics Co., Ltd., 1 Samsungjeonja-ro, Hwaseong, 18448, Republic of Korea

[3]Department of Materials Science & Engineering, Korea University, Seoul, 02841 Republic of Korea

*e-mail: bg.chae@samsung.com, eunhayo.lee@samsung.com

For the differentiation of N from Si,

Mass difference ($Si^{2+}$ – $N^{+}$) = 0.0146 Da

($Si^{2+}$ ≈ 13.9885 Da, $N^{+}$ ≈ 14.0031 Da)

Mass difference ($Si^{2+}$ – $N_2^{+}$) = 0.029 Da

($Si^{+}$ ≈ 27.977 Da, $N_2^{+}$ ≈ 28.006 Da)

A mass resolution of $m/\Delta m$ ≈ 960 (full width at half maximum (FWHM)) is theoretically required to resolve both $Si^{2+}/N^{+}$ and $Si^{+}/N_2^{+}$. However, considering peak broadening in the experimental APT mass spectra, a higher resolution—approximately 1.5 times greater of approximately $m/\Delta m$ ≈ 1,440—is likely needed.

| **Detector X × Detector Y** | **Full width half maximum (FWHM)** |
| --- | --- |
| **20 mm × 20mm** | 1,476.21 |
| **40 mm × 40mm** | 1,273.24 |
| **Total** | 1,060.43 |

**Supplementary Table. 1 Full width half maximum required to resolve N and Si as a function of detector position in the Invizo 6000.**

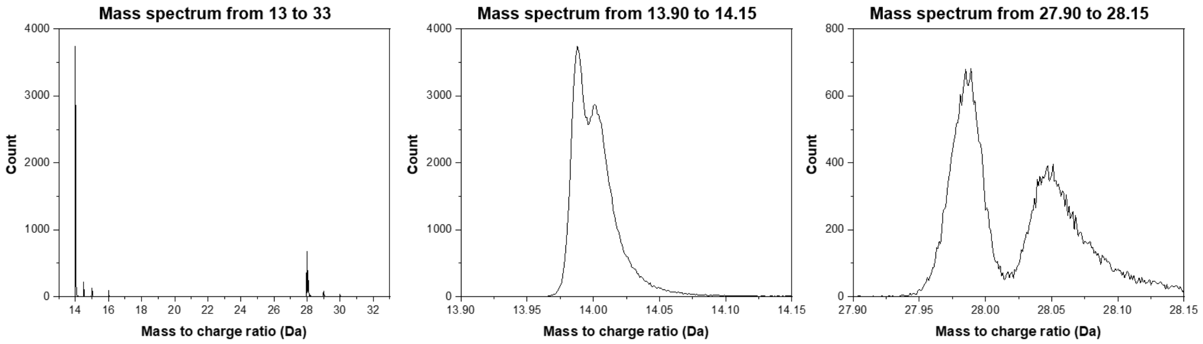


**Supplementary Fig. 1 APT mass spectra from 13 to 33 Da.** N can be distinguished from Si at both ~14 and ~28 Da, although their respective peaks partially overlap.

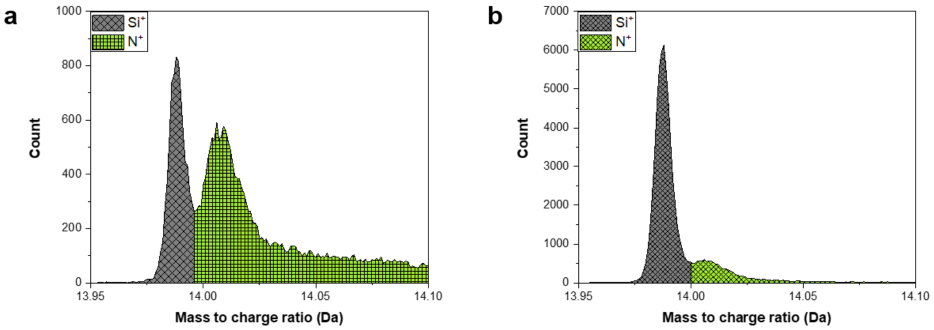


**Supplementary Fig. 2 Invizo 6000 mass spectrum of Si/$SiO_2$/SiN(25 nm) at ~14 Da.** Invizo 6000 mass spectra of $Si^{2+}$ (gray) and $N^+$(green) at ~14 Da in the structure with **a** high and **b** low N contents. $Si^{2+}$ can be distinguished from $N^+$ when the N content is significantly higher than that of Si.

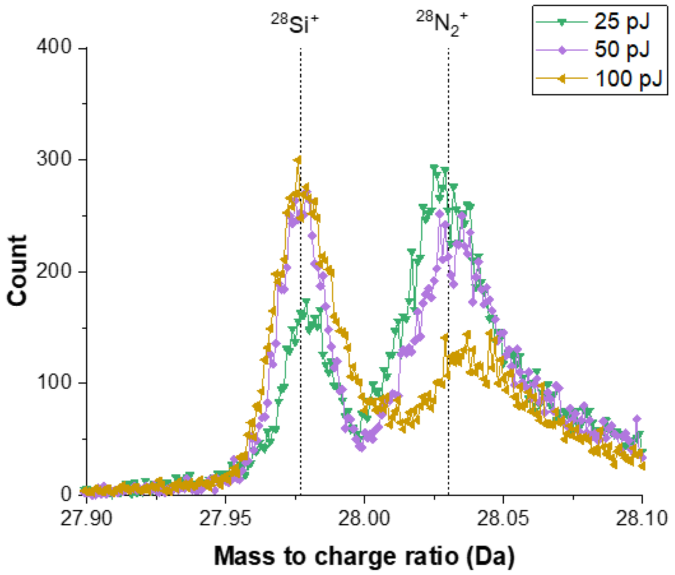


**Supplementary Fig. 3 $Si^{+}$ and $N_2^{+}$ mass peaks as a function of the laser pulse energy.** The variation in mass peak intensities is shown across a laser pulse energy range of 25 to 100 pJ. Considering both the mass peak behavior and APT success yield, 100 pJ is selected as the optimal energy for analysis.

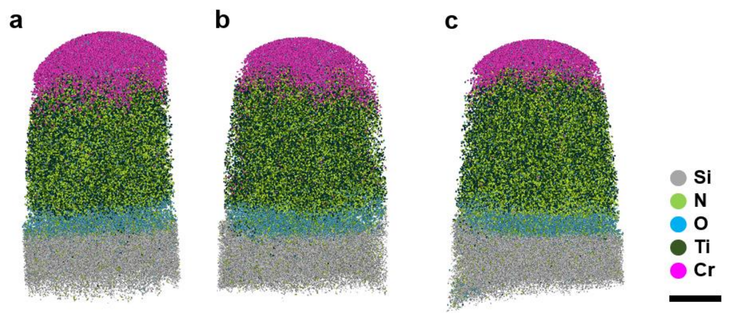


**Supplementary Fig. 4 Full reconstruction maps of the Si/$SiO_2$/SiON/TiN structure. a** without PN, **b** after PN for $t_{PN}$ followed by PNA for $t_{PNA}$, **c** and after PN for 1.5$t_{PN}$ followed by PNA for $t_{PNA}$. (Scale bars: 10 nm in **a-c**).

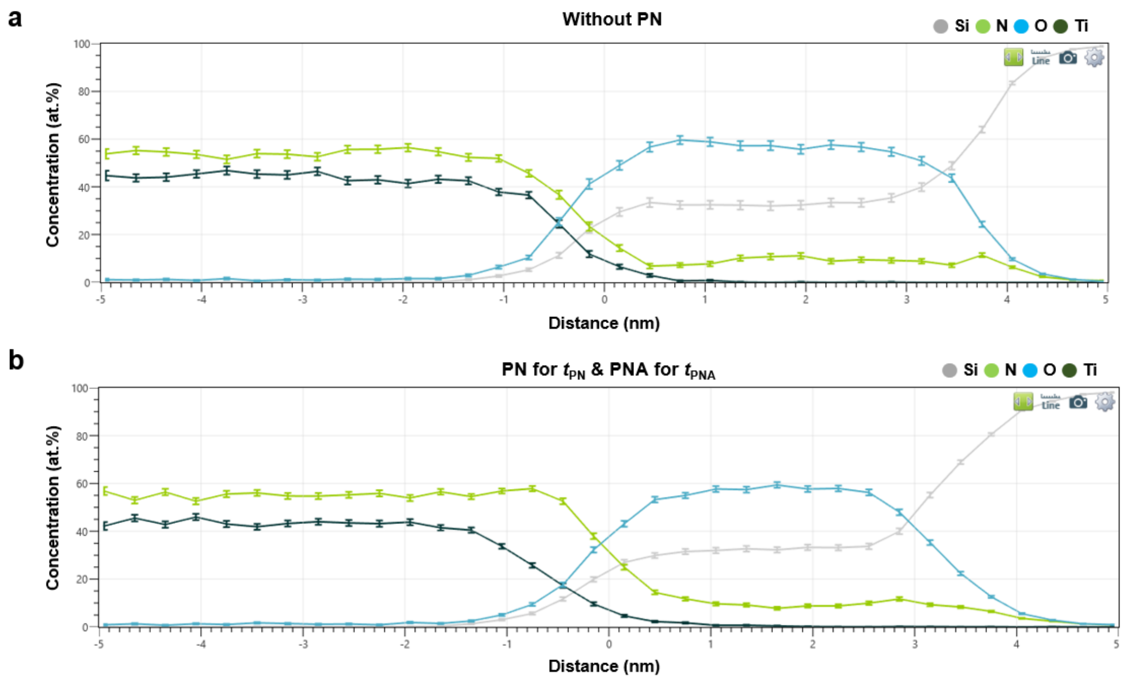


**Supplementary Fig. 5 APT proximity histogram of the Si/$SiO_2$/SiON/TiN.** APT proximity histogram from the Si 20 at.% iso-concentration surface **a** before PN and **b** after PN for $t_{PN}$ followed by PNA. The SiON layer is clearly visible after PN for $t_{PN}$ followed by PNA for $t_{PNA}$. Error bars in **a**, **b** indicate 1-sigma counting statistics.

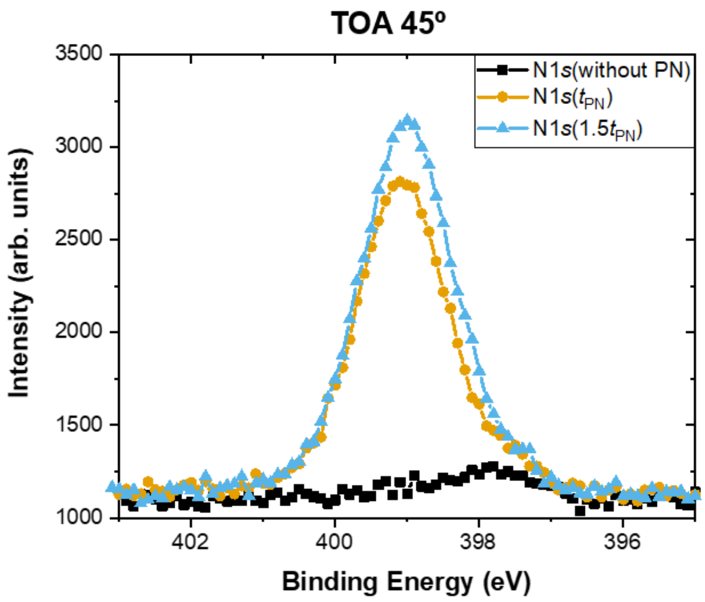


**Supplementary Fig. 6 XPS core-level spectra of the Si/$SiO_2$/SiON/TiN structure.** The N1*s* core-level spectra were obtained with a take-off angle of 45°. The N1*s* signal increased with the increasing PN time. The TiN top electrode is etched for the XPS analysis.

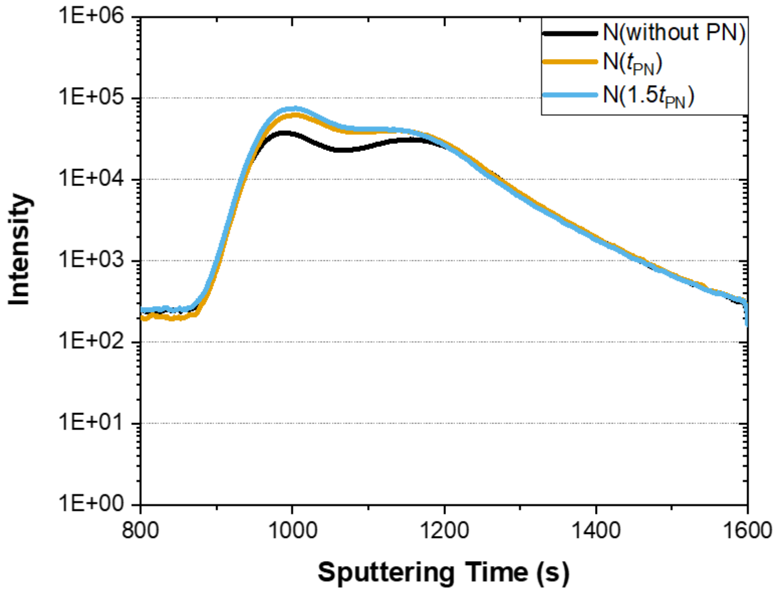


**Supplementary Fig. 7 Time of flight (ToF)-SIMS depth profile of the Si/$SiO_2$/SiON/TiN structure.** The ToF-SIMS SiN depth profile confirms the formation of the SiON layer on top of the $SiO_2$ layer. The N concentration increases with $t_{PN}$.

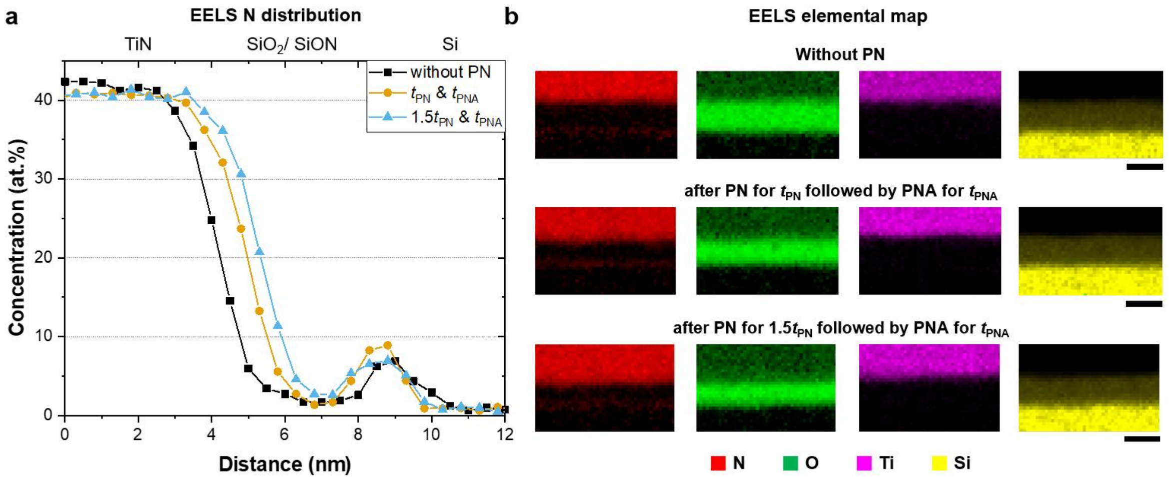


**Supplementary Fig. 8 STEM-EELS results of Si/SiO2/SiON/TiN structure.** STEM-EELS **a** profile and **b** elemental maps. A comparative analysis between samples is feasible under low e-beam dose and short EELS acquisition time per pixel. In the upper region of the gate dielectric, the N concentration is higher after 1.5$t_{PN}$ followed by $t_{PNA}$. (Scale bars: 5 nm in **b**).

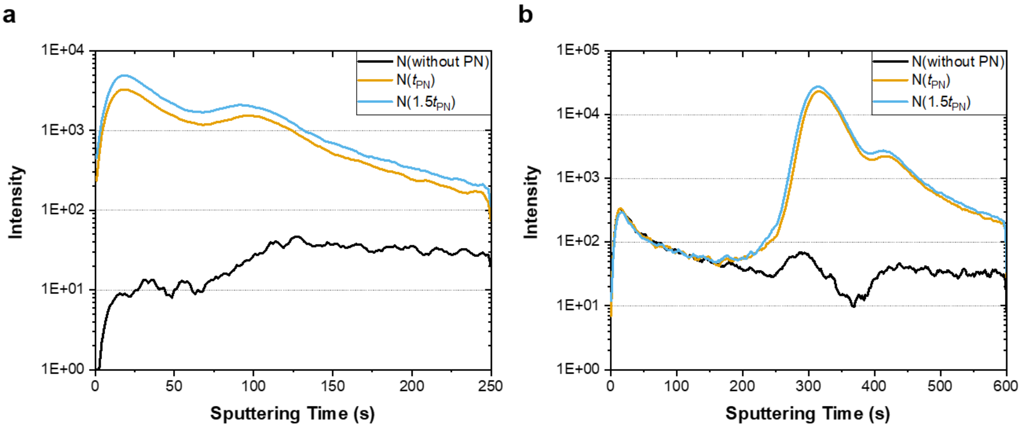


**Supplementary Fig. 9 ToF-SIMS depth profile as a function of PN process.** ToF-SIMS SiN depth profile of **a** Si/$SiO_2$/SiON and **b** Si/$SiO_2$/SiON/poly-Si structures.

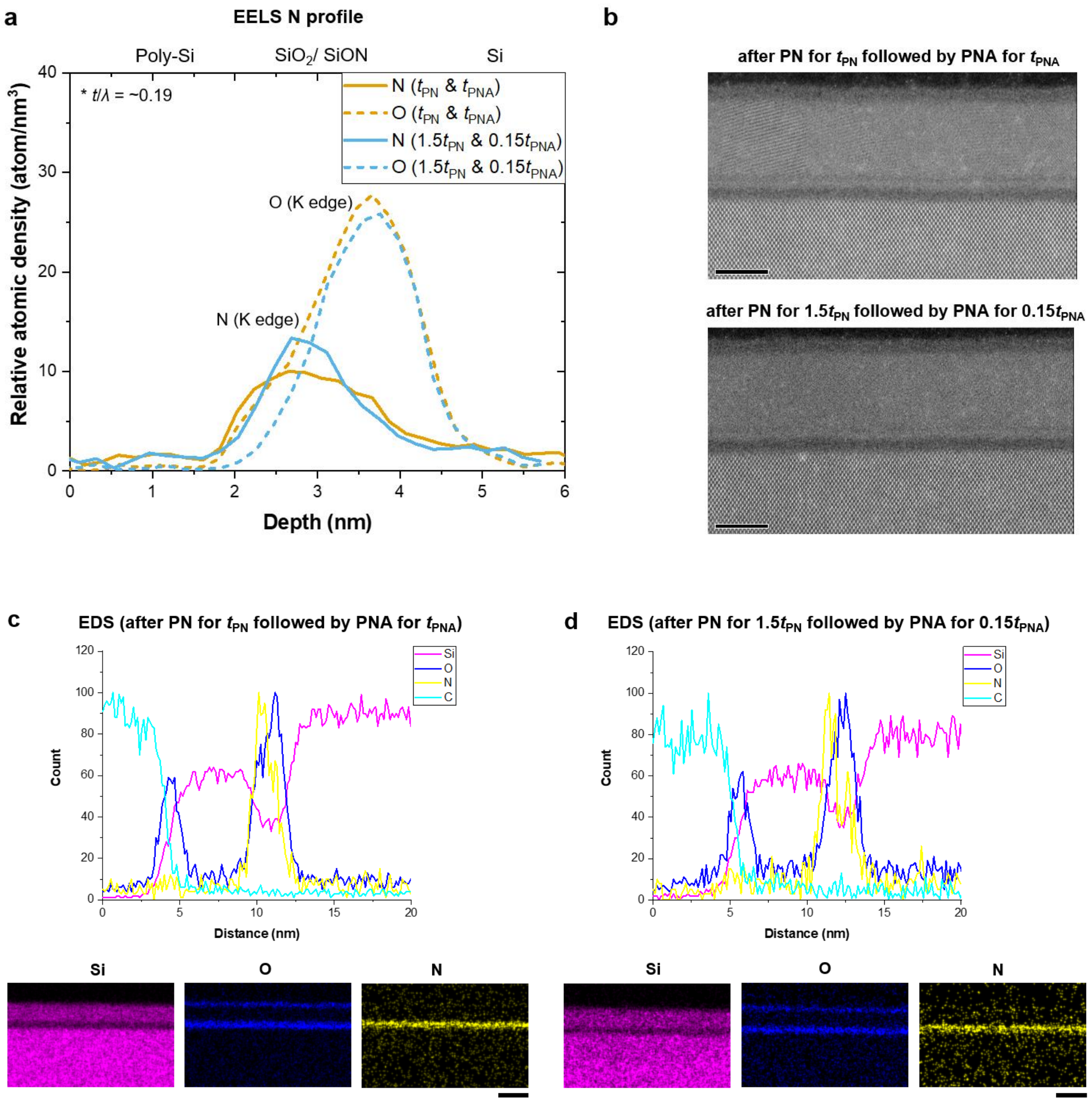


**Supplementary Fig. 10 STEM-EELS and EDS results of Si/$SiO_2$/SiON/poly-Si structure.** **a** STEM-EELS profile and **b** corresponding region of interest. A comparative analysis between samples is feasible under low e-beam dose and short EELS acquisition time per pixel. In the upper region of the gate dielectric, the N concentration is higher after 1.5$t_{PN}$ followed by 0.15$t_{PNA}$. STEM-EDS profiles and elemental maps of **c** after PN for $t_{PN}$ followed by PNA for $t_{PNA}$ and **d** after PN for 1.5$t_{PN}$ followed by PNA for 0.15$t_{PNA}$. Directly comparing the N

concentration between samples is challenging owing to the limitations of EDS in detecting N in such thin layers. (Scale bars: 5 nm in **b-d**).

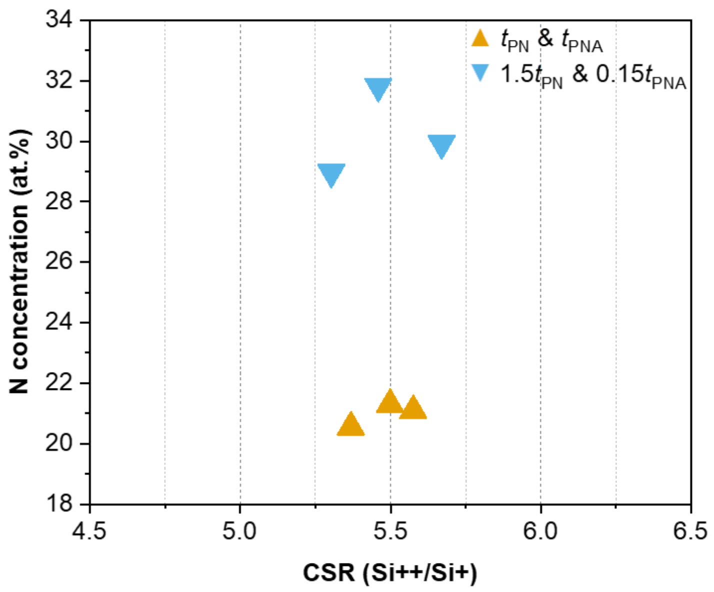


**Supplementary Fig. 11 N concentration as a function of the charge state ratio (CSR) value ($Si^{2+}/Si^{+}$).** At similar CSR values, the trends in the N concentration differ significantly depending on the PN & PNA process.

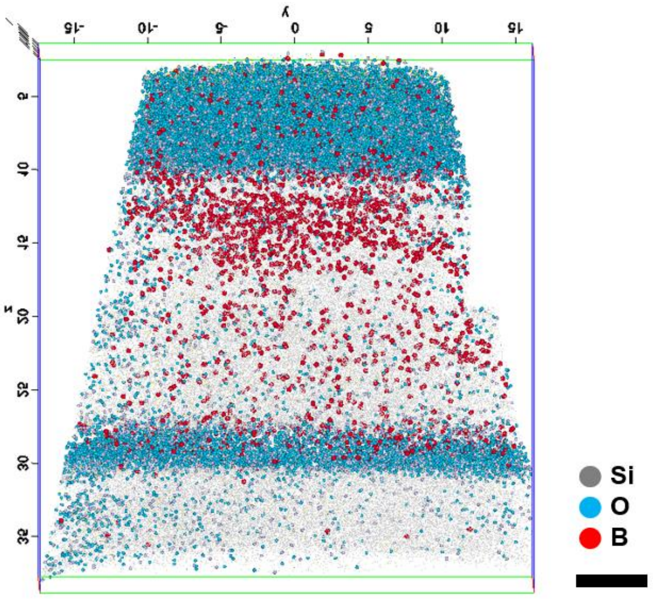


**Supplementary Fig. 12 3D ion map of the Si/$SiO_2$/SiON/B-doped poly-Si structure acquired using LEAP 5000 XR.** The SiON layer formed by the PN process is not observed in the ion map obtained using the LEAP 5000 XR. (Scale bar: 5 nm).

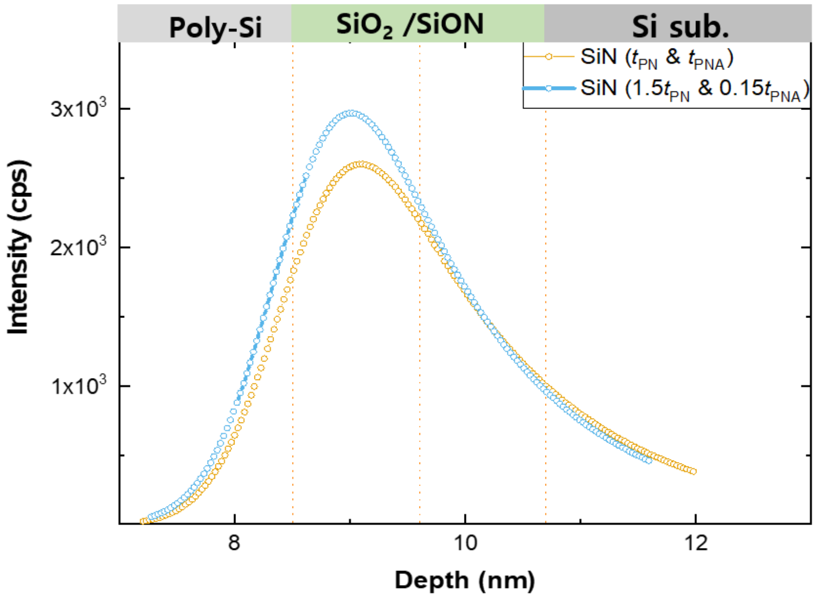


**Supplementary Fig. 13 ToF-SIMS depth profile of the Si/$SiO_2$/SiON/B-doped poly-Si structure.** The N concentration is higher but shallower after a longer PN followed by a shorter PNA.

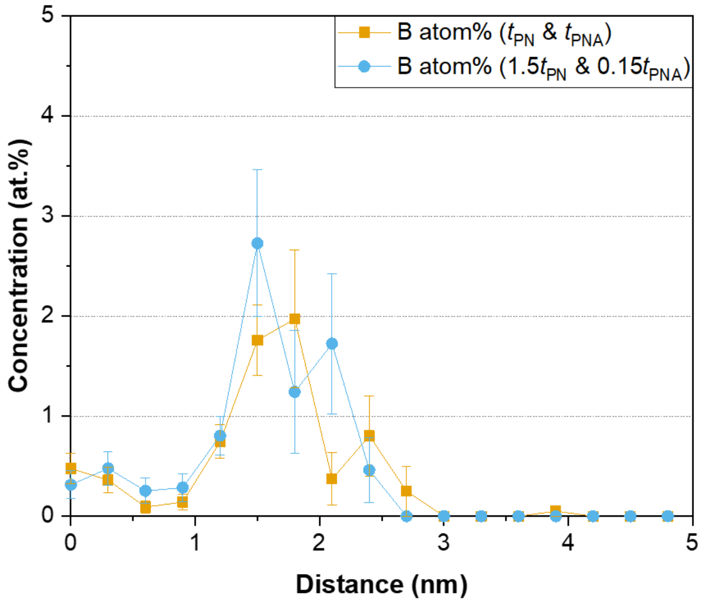


**Supplementary Fig. 14 APT B depth profile of the Si/SiO2/SiON/B-doped poly-Si structure as a function of PN and PNA process.** The diffusion of B is inhibited after a longer PN followed by a shorter PNA owing to the formation of the shallow N profile with a higher N concentration. Error bars indicate 1-sigma counting statistics.

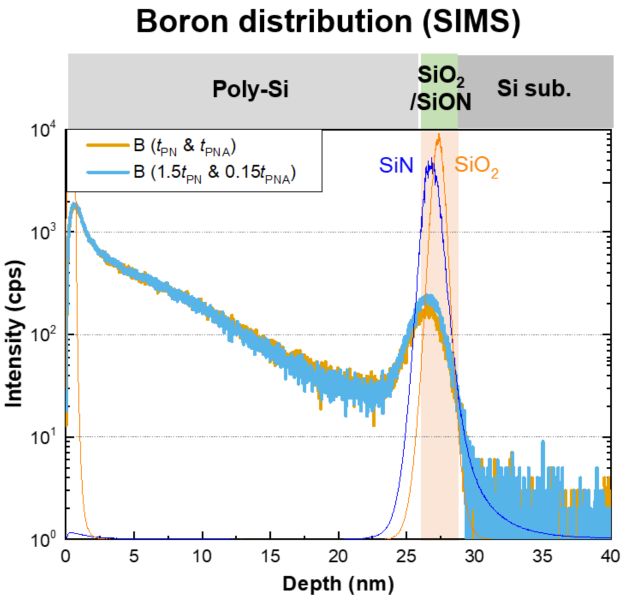


**Supplementary Fig. 15 SIMS B depth profile of the Si/SiO2/SiON/B-doped poly-Si structure as a function of PN and PNA process.** The SiON layer formed by a longer PN followed by a shorter PNA more effectively blocked B.

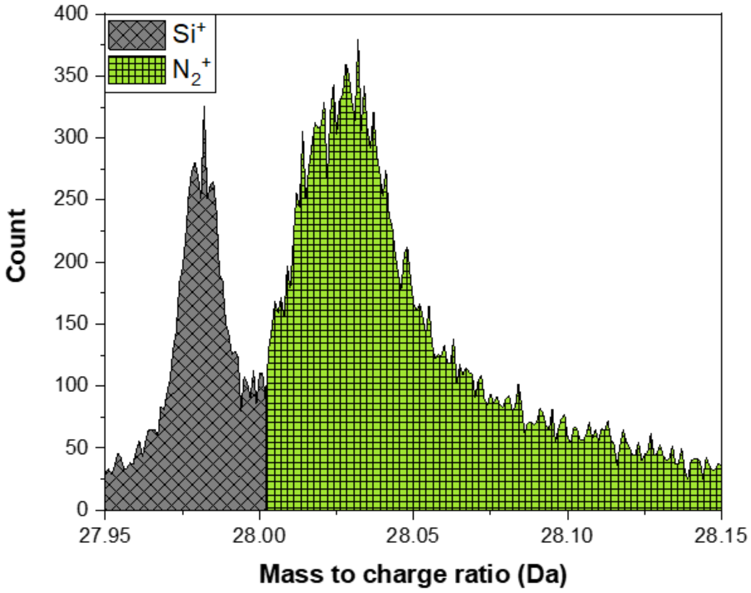


**Supplementary Fig. 16 Invizo 6000 mass spectrum of the fin-structured device.** Invizo 6000 mass spectrum of $Si^{+}$ (gray) and $N_2^{+}$(green).

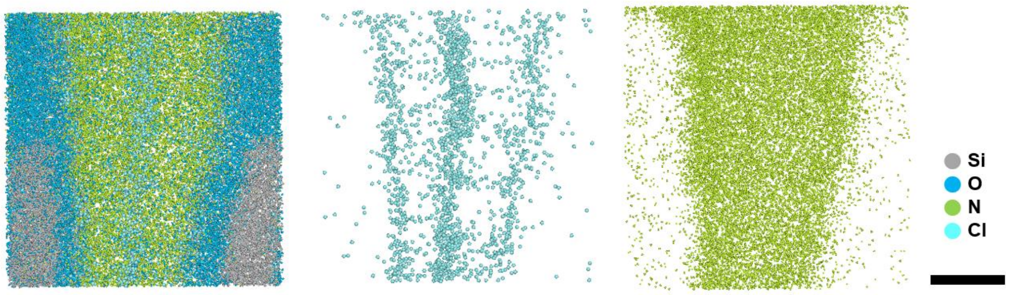


**Supplementary Fig. 17 Sliced 3D ion maps.** The density variation in the porous regions of TiN is not resolved, likely owing to the trajectory aberration. (Scale bar: 5 nm).

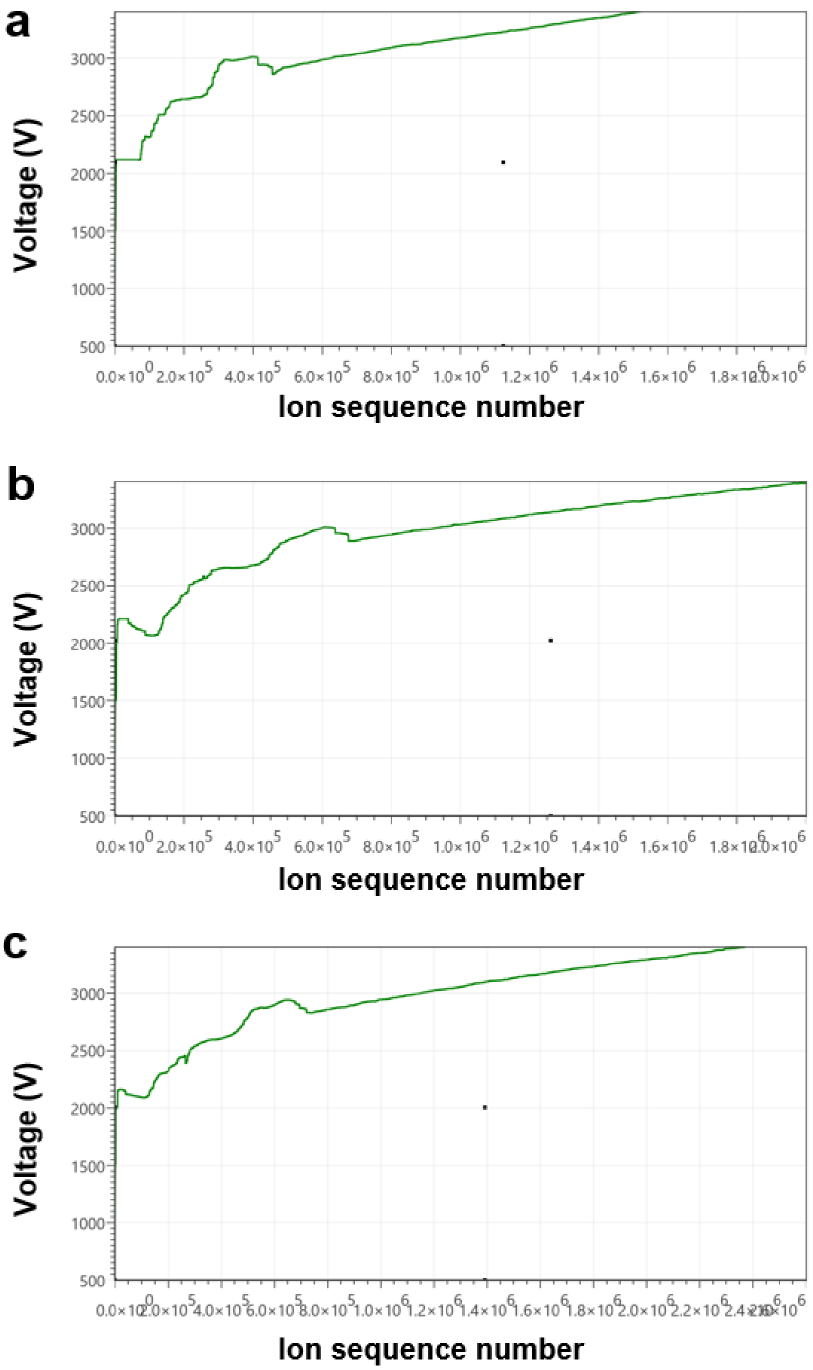


**Supplementary Fig. 18 APT voltage profiles**. Examples of APT voltage profile **a** without PN, **b** after PN for $t_{PN}$ followed by PNA for $t_{PNA}$, **c** and after PN for 1.5$t_{PN}$ followed by PNA for $t_{PNA}$. Each sample is analyzed under controlled voltage profiles.
